# Cobalt(ll) Coordination Compounds of Ethyl 4-Methyl-5-Imidazolecarboxylate: Chemical and Biochemical Characterization on Photosynthesis and Seed Germination

**DOI:** 10.1155/BCA.2005.93

**Published:** 2005

**Authors:** Beatriz King-Díaz, Josefina Montes-Ayala, Concepción Escartín-Guzmán, Silvia E. Castillo-Blum, Roberto Iglesias-Prieto, Blas Lotina-Hennsen, Norah Barba-Behrens

**Affiliations:** 1 Departamento de Bioquímica Facultad de Quimica Universidad Nacional Autónoma de .México. C. U. Coyoacán México D. F. 04510 Mexico; 2 Departamento de Química lnorgánica Facultad de Quimica Universidad Nacional Autónoma de .México. C. U. Coyoacán México D. F. 04510 Mexico; 3 Estación de lnvestigaciones Marinas “Puerto Morelos” ICMyL-UNAM. Apartado Postal 1152 Cancún Quintana Roo 77500 Mexico

**Keywords:** Ethyl 4-methyl-5-imidazolecarboxylate, Co^2+ emizco coordination compounds, cobalt(II) salts, photosynthesis, seed germination

## Abstract

In this work we present the synthesis, structural and spectroscopic characterization of Co^2+^ coordination compounds with ethyl 4-methyl-5-imidazolecarboxylate (emizco). The effects of emizco, the metal salts CoCl_2_.6H_2_O, CoBr_2_, Co(NO_3_)_2_.6H_2_O and their metal coordination compounds [Co(emizco)_2_Cl_2_], [Co(emizco)_2_ Br_2_].H_2_O, [Co(emizco)_2_ (H_2_O)_2_(NO_2_)_2_.2H_2_O were evaluated on photosynthesis in spinach chloroplasts. Seed germination and seedling growth of the monocotyledonous species *Lolium multiflorum* and *Triticum aestivum* and the dicotyledonous species *Trifolium alexandrinum* and *Physalis ixocarpa* were also assayed under the effect of the compounds and salts. The results showed that cobalt(II) salts and their emizco coordination compounds inhibit photosynthetic electron flow and ATP-synthesis, behaving as Hill reaction inhibitors. Coordination compounds are more potent inhibitors than the salts. It was found that the salts target is at the b_6_f level while the complexes targets are at Q_B_(D1)-protein and b_6_f level. The Q_B_ inhibition site was confirmed by variable chlorophyll *a* fluorescence yield. On the other hand, emizco inhibits seed germination, root and shoot development, in both weed and crop species. Cobalt(II) coordination compounds are the most effective photosynthesis inhibitors, but they are less potent than emizco in germination and seedling growth, while the metal salts are the least active of all.


### Cobalt(II) Coordination Compounds of Ethyl 4-Methyl-5-Imidazolecarboxylate: Chemical and Biochemical Characterization on Photosynthesis and Seed Germination.

Beatriz King-Díaz[1], Josefina Montes-Ayala[2], Concepción Escartín-Guzmán[2], Silvia E. Castillo-Blum[2], Roberto Iglesias-Prieto[3], Blas Lotina-Hennsen[1]* and Norah Barba-Behrens[2]*.

[1]*Departamento de Bioquímica.* [2]*Departamento de Química Inorgánica, Facultad de Química, Universidad Nacional Autónoma de México. C. U., Coyoacán, México D. F. 04510, México.* [3]*Estación de Investigaciones Marinas "Puerto Morelos", ICMyL-UNAM. Apartado Postal 1152, Cancún 77500 Quintana Roo, México.*

**ABSTRACT**

In this work we present the synthesis, structural and spectroscopic characterization of $Co^{2+}$ coordination compounds with ethyl 4-methyl-5-imidazolecarboxylate (emizco). The effects of emizco, the metal salts $CoCl_2 \cdot 6H_2O$, $CoBr_2$, $Co(NO_3)_2 \cdot 6H_2O$ and their metal coordination compounds $[Co(emizco)_2Cl_2]$, $[Co(emizco)_2Br_2] \cdot H_2O$, $[Co(emizco)_2(H_2O)_2](NO_3)_2 \cdot 2H_2O$ were evaluated on photosynthesis in spinach chloroplasts. Seed germination and seedling growth of the monocotyledonous species *Lolium multiflorum* and *Triticum aestivum* and the dicotyledonous species *Trifolium alexandrinum* and *Physalis ixocarpa* were also assayed under the effect of the compounds and salts. The results showed that cobalt(II) salts and their emizco coordination compounds inhibit photosynthetic electron flow and ATP-synthesis, behaving as Hill reaction inhibitors. Coordination compounds are more potent inhibitors than the salts. It was found that the salts target is at the $b_6f$ level while the complexes targets are at $Q_B$(D1)-protein and $b_6f$ level. The $Q_B$ inhibition site was confirmed by variable chlorophyll *a* fluorescence yield. On the other hand, emizco inhibits seed germination, root and shoot development, in both weed and crop species. Cobalt(II) coordination compounds are the most effective photosynthesis inhibitors, but they are less potent than emizco in germination and seedling growth, while the metal salts are the least active of all.

**Key words:** Ethyl 4-methyl-5-imidazolecarboxylate, $Co^{2+}$ emizco coordination compounds, cobalt(II) salts, photosynthesis, seed germination.

* Tel/fax 52(55)56-22-38-10, email: norah@servidor.unam.mx

### INTRODUCTION

It is known that imidazole derivatives are extensively used in pharmaceutical /1/ and agrochemical industries /2,3/. Ethyl 4-methyl-5-imidazolecarboxylate (emizco) derivatives present antiviral /4/ and herbicidal /5/ activities. Most commercial herbicides are organic synthetic compounds with a wide variety of structures and with different targets, selectivity, mode of action, and weed spectrum. In order to exert toxicity, all pre-emergence herbicides (or post-emergence) must be absorbed into the root (or foliage) and move to the site of action, where they must be present at an active concentration and a proper toxic form for a long-enough period /6/. Any factor that interferes with this sequence may account for differential selectivity or sensitivity of herbicides between species among herbicides. Properties, or spatial distribution of herbicides functional groups are also important factors that may affect their selectivity.

It is known that the phytotoxicity of the organic compound is either enhanced or decreased upon coordination with metal ions /7/. The primary interest in our screening program is directed to compounds that affect seed germination, seedling root and shoot development or energy metabolism. We have previously studied the effects of transition metal coordination compounds on different photosynthetic activities. $Ni^{2+}$ coordination compounds of emizco and nickel salts inhibited thylakoid electron transport chain at two different targets, being nickel salts less potent as Hill reaction inhibitors. Emizco was inactive on photosynthetic activities /8/. Continuing with this work, we extended our research to the effect of cobalt salts and their emizco coordination compounds on photosynthesis and germination.

Some transition metal ions participate in a great variety of biological roles, among them, as essential components of several enzymes /9/. These elements are plant micronutrients affecting their growth and metabolism /9,10/. For example, cobalt sulphate decreased seed germination of *Pinus sylvestris* /11/ and *Nicotiana tabacum* /12/. This metal ion stimulates yeast mitochondria respiration when α-keto glutarate is employed, but it was irreversibly inhibited in the presence of succinate. Pre-incubation of mitochondria with cobalt sulphate inhibited respiration and oxidative phosphorylation /13/. The effect of $Co^{2+}$ on photosynthesis is controversial. Tripathy *et al* /14/ reported that cobalt inhibits the oxidizing side of the PSII reaction centre. Furthermore, it interacts with $Q_B$ site /10,15/. Here we report the synthesis and characterization of emizco cobalt(II) coordination compounds. We also investigated the effect on photosynthesis, seed germination, seed respiration and root and shoot development of emizco, its $Co^{2+}$ complexes, and cobalt(II) salts.

### EXPERIMENTAL

### Materials

All chemicals were reagent grade: solvents (Merck); ethyl 4-methyl-5-imidazolecarboxylate (emizco) (Aldrich); $CoCl_2 \cdot 6H_2O$, $CoBr_2$, $Co(NO_3)_2 \cdot 6H_2O$ (J. T. Baker) were used without further purification. Sorbitol, sucrose, tricine, KCN, KCl, $MgCl_2$, KOH, $NH_4Cl$, $[Fe(CN)_6]$, Methyl viologen (MV), N-(2-hydroxyethyl)piperazine-N'-(2-ethanesulfonic acid) (HEPES), 3-(3,4-dichlorophenyl)-1,1-dimethylurea (DCMU), 2,6-dichlorophenol indophenol (DCPIP), 2,5-dibromo-6-isopropyl-3-methyl-1,4-benzoquinone

(DBMIB), sodium silicomolybdate (SiMo) and reduced tetramethyl-p-benzoquinone ($TMQH_2$) were purchased from Sigma-Aldrich.

### Synthesis of emizco coordination compounds

$[Co(emizco)_2Cl_2]$, $[Co(emizco)_2Br_2]\cdot H_2O$ and $[Co(emizco)_2(H2O)_2](NO_3)_2\cdot 2H_2O$, were prepared by adding the appropriate salt (0.5 mmol) dissolved in 15 $cm^3$ of methanol to emizco (1 mmol), (1:2 molar ratio) dissolved in 15 $cm^3$ of hot methanol, and refluxed for five hours. The solution was allowed to stand at room temperature for two to six weeks. The coordination compounds were characterized by different spectroscopic and chemical techniques (IR, electronic absorption spectroscopy, elemental analyses, thermogravimetric studies and magnetic susceptibility).

### $[Co(emizco)_2Cl_2]$

A solution of emizco (0.1542 g, 1.0 mmol) in hot methanol (15 $cm^3$) was added to a solution of $CoCl_2\cdot 6H_2O$ (0.24 g, 0.5 mmol) in hot methanol (15 $cm^3$). The mixture was heated under reflux for 5 h. A navy blue microcrystalline precipitate was obtained by slow evaporation of the solvent and it was vacuum filtered. Anal. Calc. for $C_{14}H_{20}O_4Cl_2N_4Co$: C, 38.38%; H, 4.60%; N, 12.79%. Found: C, 37.66%; H, 4.57%; N, 12.86%. IR (KBr, $cm^{-1}$): 1730, 1690 ν(C=O), 1600 ν(C=N).

### $[Co(emizco)_2Br_2]\cdot H_2O$

A solution of emizco (0.1542 g, 1.0 mmol) in hot methanol (15 $cm^3$) was added to a solution of $CoBr_2$ (0.238 g, 0.5 mmol) in hot methanol (15 $cm^3$). The mixture was heated under reflux for 5 h. A navy blue microcrystalline precipitate was obtained by slow evaporation of the solvent and it was vacuum filtered. Anal. Calc. for $C_{14}H_{22}O_5Br_2N_4Co$: C, 30.80%; H, 4.07%; N, 10.28%. Found: C, 29.20%; H, 3.17%; N, 10.80%. IR (KBr, $cm^{-1}$): 1736, 1690 ν(C=O), 1599 ν(C=N).

### $[Co(emizco)_2(H_2O)_2](NO_3)_2\cdot 2H_2O$

A solution of emizco (0.1542 g, 1.0 mmol) in hot methanol (15 $cm^3$) was added to a solution of $Co(NO_3)_2\cdot 6H_2O$ (0.291 g, 0.5 mmol) in hot methanol (15 $cm^3$). The mixture was heated under reflux for 5 h. A pink microcrystalline precipitate was obtained by slow evaporation of the solvent and it was vacuum filtered. Anal. Calc. for $C_{14}H_{28}O_{14}N_6Co$: C, 29.85%; H, 5.00%; N, 14.92%. Found: C, 28.75%; H, 4.22%; N, 15.81%. IR (KBr, $cm^{-1}$): 1676 ν(C=O), 1600(sh) ν(C=N), 1384 ν($NO_3^-$).

### Stability of the coordination compounds

This study was carried out using $10^{-3}$ M aqueous solutions of $[Co(emizco)_2Cl_2]$, $[Co(emizco)_2Br_2]\cdot H_2O$ and $[Co(emizco)_2(H_2O)_2](NO_3)_2\cdot 2H_2O$, by UV-visible absorption spectroscopy. Experiments using different buffers (such as HEPES and Tricine) varying the concentration of the complexes were performed in order to know if the coordination compounds under study interacted with the buffers. The buffer concentration was varied from 20 to 40 mM, for two different pH values: 7.0 and 8.0.

### Chloroplasts isolation and chlorophyll determination

Intact chloroplasts were isolated from market spinach leaves (*Spinacea oleracea L.*) as previously described /16,17/, and suspended in 400 mM sucrose, 10 mM KCl, 5 mM $MgCl_2$ and 30 mM tricine buffer (pH 8.0 with the addition of KOH). They were stored as a concentrated suspension in the dark for 1 hour at 0°C. Intact chloroplasts were lysed to yield free thylakoids previous to each experiment by incubating them in 100 mM sorbitol, 0.5 mM KCN, 10 mM KCl, 5 mM $MgCl_2$ and 20 mM HEPES-KOH buffer (pH 8.0). Chlorophyll was determined according to the reported method /18/.

### Physical Measurements

A FTIR spectrometer (Perkin Elmer 599 B) was used for obtaining spectra of solid samples in KBr pellets (4000 – 400 $cm^{-1}$). The UV-Vis spectra (diffuse reflectance and solution, 40000 – 4000 $cm^{-1}$) were recorded on a Cary-5E (Varian) spectrometer. Elemental analyses were carried out with a Fisons EA 1108 analyser. Magnetic susceptibility measurements at room temperature, of powdered samples, were recorded on a Johnson-Matthey DG8 5HJ balance using the Gouy's method.

### Measurement of electron transport and ATP-synthesis

ATP-synthesis was determined titrimetrically using an Orion Mod. 8103 Ross microelectrode connected to a Model 12 Corning potentiometer, with expanded scale as reported by Dilley /19/. The ATP-synthesis medium contained 100 mM sorbitol, 0.5 mM KCN, 10 mM KCl, 5 mM $MgCl_2$, 50 μM MV, and 1 mM HEPES-KOH buffer (pH 8.0).

Photosynthetic non-cyclic electron transport was monitored with an YSI (Yellow Springs Instrument) Model 5300 oxygen monitor using a Clark electrode in a temperature-regulated flask at 20 °C. The reaction medium was similar to that for ATP-synthesis but HEPES concentration was changed to 15 mM at the same pH. 20 μg/mL chloroplasts were added (whole electron chain transport). The sample was illuminated for 1 minute in the presence or absence of 6 mM $NH_4Cl$ /16,20/.

PSII was measured by photo-reduction of DCPIP monitored polarographycally by $O_2$ evolution. The reaction medium for assaying PSII activity contained the same whole electron chain transport medium ($H_2O \rightarrow MV$) above mentioned, without methylviologen but in the presence of 1 μM DBMIB, 100 μM DCPIP, 300 μM $[Fe(CN)_6]$ and 6 mM $NH_4Cl$.

PSI electron transport was determined in a similar form to non-cyclic electron transport. The following reagents were added: 100 μM DCPIP, 300 μM ascorbate, 10 μM DCMU and 6 mM $NH_4Cl$ /21/.

Electron transport chain of the partial reactions of the PSII and PSI were measured using specific inhibitors: 10 μM DCMU, 1 μM DBMIB, 30 mM KCN, and the following electron donors and acceptors:

100 μM SiMo, 100 μM DCPIP and 50 μM MV /21/.

### Chlorophyll *a* fluorescence assays

Chl *a* fluorescence induction curves of freshly lysed chloroplasts were measured at room temperature using a PEA (Plant Efficiency Analyser) fluorometer (Hansatech UK), as described /8, 22/. Aliquots of dark-adapted thylakoids containing 15 $\mu g/cm^3$ of Chl *a* were suspended in the electron transport medium and transferred with a dot-blot apparatus (Bio Rad USA) to filter paper. The thylakoids were immediately transferred to vials containing 3 $cm^3$ of solutions of the tested compounds, which contained different concentrations and were incubated for 5 min in the dark.

### Seed germination bioassay

Uniform size seeds were selected. For the germination bioassays, 40 wheat seeds (*Triticum aestivum)* and 100 seeds of *Physalis ixocarpa, Lolium multiflorum* and *Trifolium alexandrinum*, were set into 10 cm Petri dishes containing a 10 cm filter paper and 10 $cm^3$ of a solution containing the test compound, or 10 $cm^3$ deionised water as control /23/. The seeds were imbibed in water, or an aqueous solution of the ligand, cobalt salts, or coordination compounds, at different concentrations. They were let to stand for 5 days (3 days for germination and 2 more days for growth) in the dark at 28° C. Emergence of radicle from the seed was taken as indication of germination.

### Seed respiration bioassays

Experiments on respiration followed the same procedure as that for germination bioassay. However, the seed respiration was measured as $O_2$ uptake using a Clark type electrode attached to a Yellow Spring Instrument (YSI) model 5300 oxymeter. The current generated during $O_2$ reduction to water was converted to voltage, and the signal recorded on a Gilson chart recorder. The current was stoichiometrically related to the oxygen consumed at the cathode.

The seeds were imbibed in water on Petri dishes, and an aqueous solution of the ligand, cobalt salts, or coordination compounds, was added at concentrations ranging from 100 to 400 μM. The Petri dishes were placed in the respiration chamber of an oxymeter. $O_2$ uptake was monitored for 3 min and then the seeds were discarded. The oxygen consumption rate was calculated and reported in nano atom $O_2$ x $h^{-1}$ x $seed^{-1}$. Temperature was kept at 20°C.

### RESULTS AND DISCUSSION

The IR spectrum of emizco shows absorption bands corresponding to the stretching modes ν(C=O), $\nu_{as}$(C-O-C), $\nu_s$(C-O-C) of the ester functional group at 1698, 1320, and 1178 $cm^{-1}$ respectively, and for the ν(C=N) at 1510 $cm^{-1}$. In all cobalt coordination compounds ν(C=N) shifts to higher frequencies (1514-1600

$cm^{-1}$), due to the coordination of the imidazole nitrogen atom N3 to the metal ion. In the spectra of $[Co(emizco)_2Cl_2]$ and $[Co(emizco)_2Br_2]\cdot H_2O$ the ν(C=O) band is splitted and shifted to higher energy (1730 and 1690 $cm^{-1}$) indicating that the ligands are not coordinated to $Co^{2+}$ by the ester oxygen atoms, only by the heterocyclic nitrogen, as has been previously observed for analogous cobalt(II) imidazolic compounds, [24,25]. On the other hand, for $[Co(emizco)_2(H_2O)_2](NO_3)_2\cdot 2H_2O$, the ester bands were shifted to *ca.*1672, 1328 and 1214 $cm^{-1}$ respectively, indicating that the two ligands are coordinated to the metal ion in a bidentate mode, through the oxygen of the ester group and the imidazolic nitrogen. There were observed vibration bands at 1384 and 1094 $cm^{-1}$ indicating the presence of ionic $NO_3^-$.

The blue $Co^{2+}$ complexes $[Co(emizco)_2Cl_2]$ and $[Co(emizco)_2Br_2]\cdot H_2O$ show the expected magnetic moments (4.52 and 4.51 BM). Their electronic spectra (diffuse reflectance) are typical of tetrahedral complexes, with transitions $\nu_2$ $^4T_1(F) \leftarrow {}^4A_2(F)$ and $\nu_3$ $^4T_1(P) \leftarrow {}^4A_2(F)$ at 1302 and 600 nm for the chloro compound, and for the bromo complex at 1295 and 610 nm. This difference between the spectra of both compounds indicates that the halides are coordinated (Scheme 1). The electronic spectrum of $[Co(emizco)_2(H_2O)_2](NO_3)_2\cdot H_2O$ shows bands as expected for an octahedral complex, $\nu_1$ 1075 nm, $\nu_2$ 600 (sh) and $\nu_3$ at 500 nm, corresponding to $^4T_{2g}(F) \leftarrow {}^4T_{1g}(F)$, $^4A_{2g}(F) \leftarrow {}^4T_{1g}(F)$, $^4T_{1g}(P) \leftarrow {}^4T_{1g}(F)$ and its magnetic moment is as expected.

From its chemical and spectroscopic characterization, an octahedral geometry is proposed for $[Co(emizco)_2(H_2O)_2](NO_3)_2\cdot 2H_2O$, similar to that of the X-ray structure of its $Ni^{2+}$ analogue /8/. Where emizco behaves as a chelate ligand, bonded to the metal ion through the oxygen atom from the ester group and the imidazolic nitrogen, and two water molecules coordinated to the metal atom (Scheme 1).

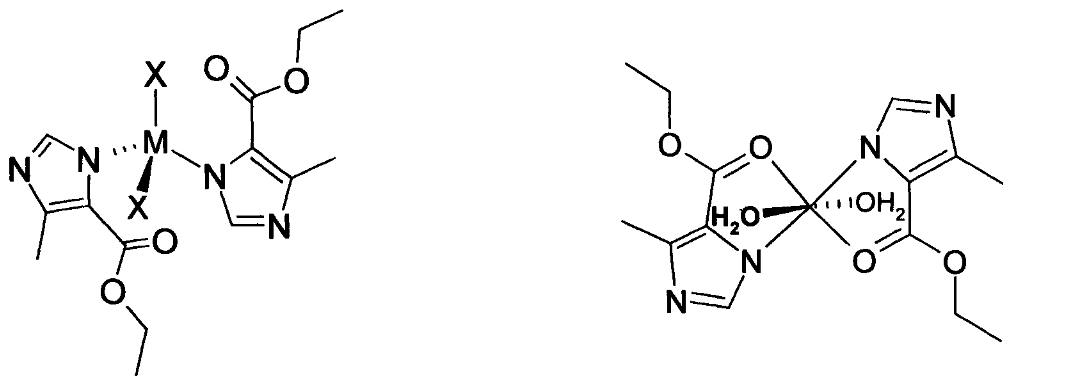

**Scheme 1.** $[Co(emizco)_2X_2]$, where X = $Cl^-$, $Br^-$ and $[Co(emizco)_2(H_2O)_2]^{2+}$

### Solution characterization of the coordination compounds

The tetrahedral $[Co(emizco)_2Cl_2]$ and $[Co(emizco)_2Br_2]\cdot H_2O$ compounds become hexa-coordinated in aqueous solution, with an octahedral arrangement, adding two coordinated water molecules. The hexa-coordinated complex $[Co(emizco)_2(H_2O)_2](NO_3)_2\cdot 2H_2O$ conserves its octahedral geometry in aqueous solution, as indicated by its UV-visible spectrum in solution [26]. The emizco ligand remains coordinated in a bidentate mode through the imidazolic nitrogen and the oxygen atom. For the chloro and bromo compounds it can be inferred, from the UV-visible absorption spectra, that water molecules substituted the halides, Fig. 1.

It was shown that the buffer did not coordinate to the metal ion in any of the compounds (Fig. 1). The UV-visible spectra of the compounds remained unchanged for solutions of increasing buffer concentration (Fig. 1). Emizco remains bonded to the metal ion in aqueous solution for at least three days, as indicated from kinetic studies. Therefore, the active species in all the experiments contains the emizco ligand coordinated to $Co^{2+}$.

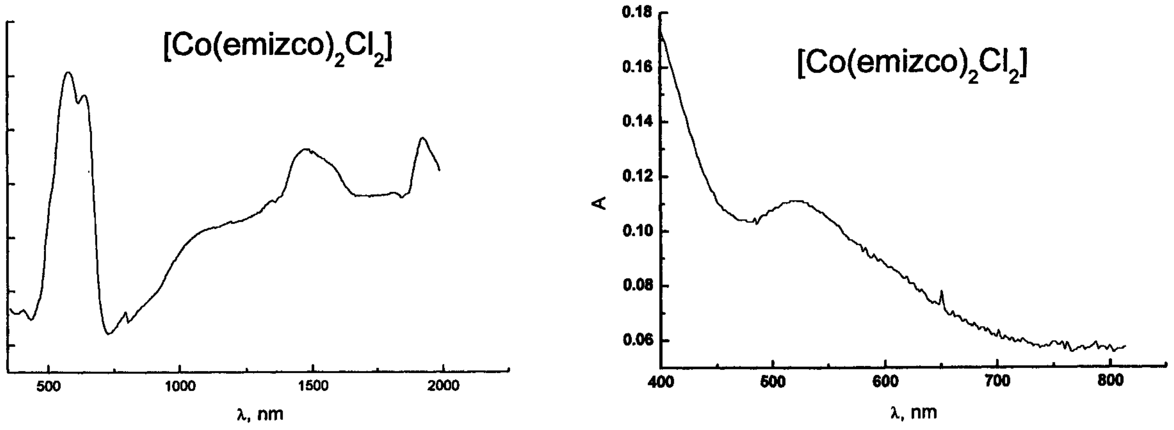

**Fig. 1:** UV-Visible absorption spectra of $[Co(emizco)_2Cl_2]$. Left: diffuse reflectance spectrum, right: $6 \times 10^{-3}$ M buffered aqueous solution in $6 \times 10^{-2}$ M HEPES, pH 8.0.

### ATP-synthesis and electron transport chain

The effect of emizco, the cobalt(II) salts and their coordination compounds on ATP-synthesis and on the electron transport chain was determined on spinach thylakoids. The results indicated that emizco has no activity. Cobalt(II) salts and their coordination compounds behave as Hill reaction inhibitors, as they inhibited basal, phosphorylating and uncoupled electron flow; and ATP-synthesis in a concentration-dependent manner (Fig. 2). Coordination compounds are more potent inhibitors than the salts. Fig. 2 shows the effect of $CoCl_2$ (2A) and its coordination compound $[Co(emizco)_2Cl_2]$ (2B) on ATP-synthesis and basal, phosphorylating and uncoupled electron transports from $H_2O$ to MV. The $I_{50}$ values for the uncoupled electron transport were 50 and 400 μM respectively.

In order to localise the inhibition site of the salts and the coordination compounds on the electron transport chain, the effect on photosystems II and I was measured on partial photosynthesis reactions. Artificial electron donors and acceptors are used to study partial reactions of the electron transport chain, as shown in Fig. 3.

The cobalt(II) salts inhibited the electron flow measured from $TMQH_2$ to MV (Table 1) and they did not affect the electron flow of PSII, measured from $H_2O$ to DCPIP, neither PSI, measured from $DCPIPH_2$. These results indicate that the target is at the $b_6f$ level.

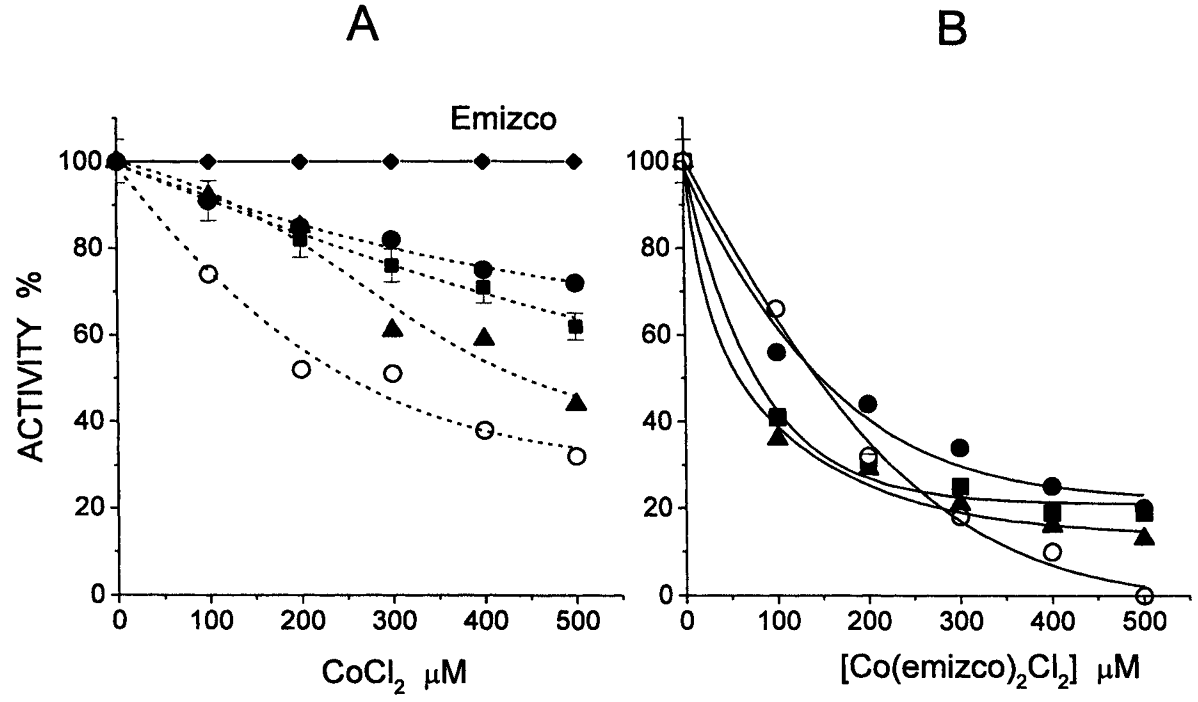

**Fig. 2:** A. Effect of $CoCl_2$ on ATP-synthesis (O) and photosynthetic electron transport from water to MV on freshly lysed spinach chloroplasts: basal (■), phosphorylating (●) and uncoupled (▲) electron transport rate. Emizco ligand did not present any effect (◆). Figure 2B. Effect of [Co(emizco)$Cl_2$] on ATP-synthesis (O) and photosynthetic electron transport from water to MV on freshly lysed spinach chloroplasts: basal (■), phosphorylating (●) and uncoupled (▲) electron transport rate. Emizco ligand did not present any effect (◆). Control average rates are 311, 640, 1346 μequiv $e^-$/mg chl per h., for basal, phosphorylating and uncoupled electron flows, respectively, and 1117 μM Pi/mg chl per h for ATP-synthesis. Each curve is the average of three replicates.

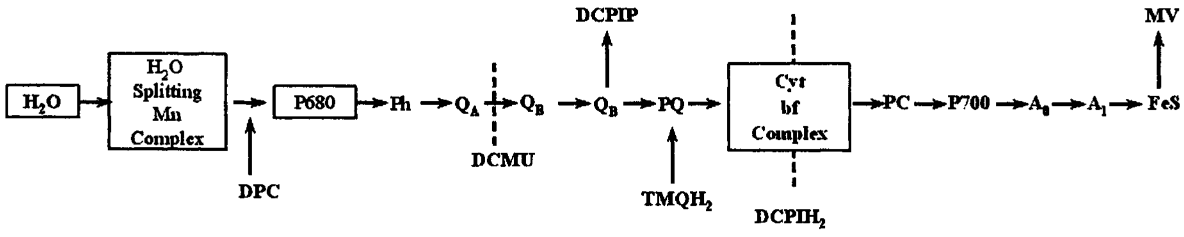

**Fig. 3:** Model of photosynthetic electron transport in isolated chloroplasts showing sites of electron donation and acceptance (arrows) and sites of inhibition (broken lines) by commercial inhibitors.

On the other hand, the coordination compounds inhibited PSII and electron flow from $TMQH_2$ to MV, but did not inhibit PSI or electron transport from water to SiMo (Table 1); indicating that their targets are at $Q_B$-protein and at $b_6f$ level. The $Q_B$ inhibition site was confirmed by variable chlorophyll *a* fluorescence yield. An increase in relative variable fluorescence yield as a function of the cobalt complexes concentration was indicative of a loss in $Q_A^-$ re-oxidation capacity (Fig. 4). The salts and the complexes have one common inhibition site located at $b_6f$, while the coordination compounds have a second target at $Q_B$.

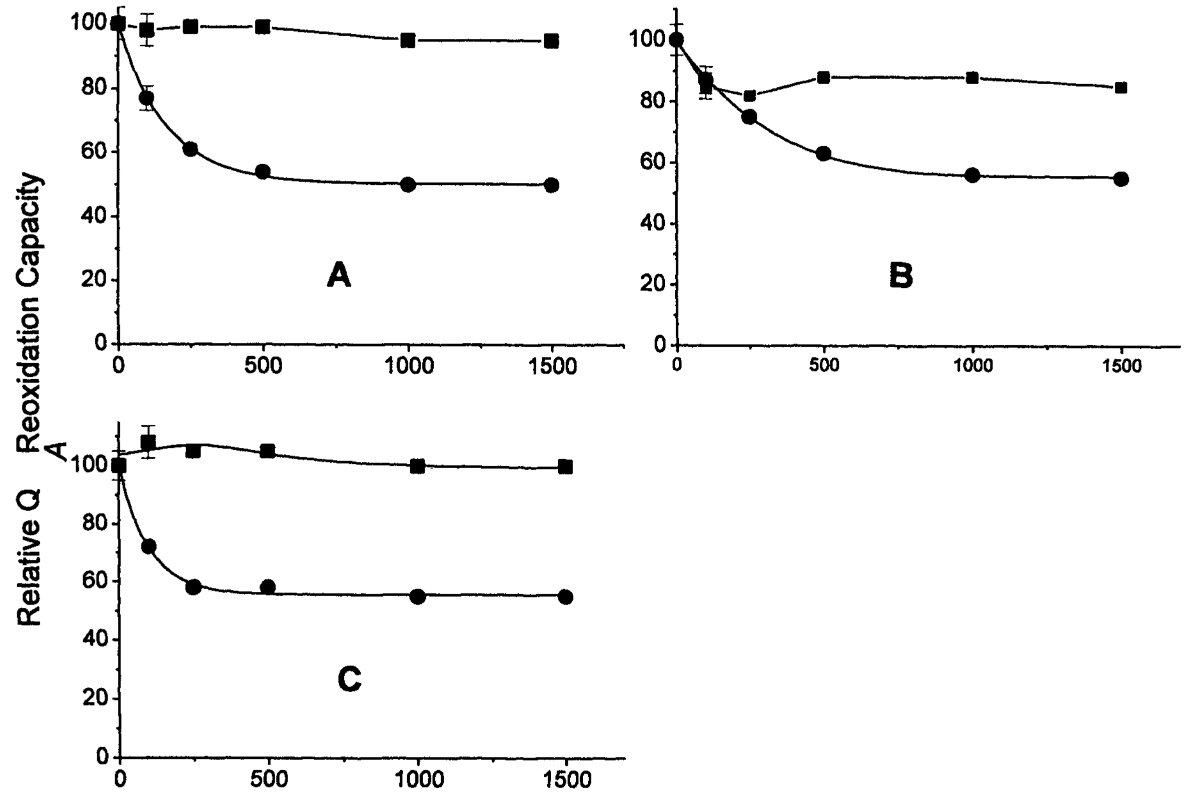

**Fig. 4:** Relative variable fluorescence, F(V), corresponding to the electron transfer from $Q_A$ to $Q_B$ as a function of concentration: (A) $CoCl_2$ $6H_2O$ (■) and $[Co(emizco)_2Cl_2]$ (●); (B) $CoBr_2$ (■) and $[Co(emizco)_2Br_2]$ $H_2O$; (●) (C) $Co(NO_3)_2$ $6H_2O$ (■) and $[Co(emizco)_2(H_2O)_2](NO_3)$ $2H_2O$ (●).

### Chlorophyll a fluorescence measurements

The reduction of the relative $Q_A^-$ re-oxidation capacity of thylakoids on addition of coordination compounds was significantly concentration dependent (Fig. 4); while cobalt(II) salts showed no effect. Inhibition of PSII electron transport activity, from $H_2O$ to DCPIP, was determined by polarography. A correlation was found between the accumulation of $Q_A^-$ and the inhibition on PSII (Table 1 and Fig. 4). These observations strongly suggest that the target site of the coordination compounds is located at the acceptor side of the PSII at the $Q_B$-protein.

**Table 1**

Effect of cobalt(II) salts and emizco coordination compounds at 500 μM on uncoupled electron transport rate, PSII, PSI and partial reactions.

| Compound | Uncoupled | | PIS | | PSI | | $TMQH_2$ to MV | | $H_2O$ to SiMo | |
|---|---|---|---|---|---|---|---|---|---|---|
| | *a* | *b* | *a* | *b* | *a* | *b* | *a* | *b* | *a* | *b* |
| Control | 1346 | 100 | 662 | 100 | 1750 | 100 | 1042 | 100 | 242 | 100 |
| $CoCl_2{\cdot}6H_2O$ | 538 | 40 | 649 | 98 | 1750 | 100 | 375 | 36 | 242 | 100 |
| $[Co(emizco)_2Cl_2]$ | 202 | 15 | 238 | 36 | 1750 | 100 | 375 | 36 | 242 | 100 |
| $CoBr_2$ | 538 | 40 | 569 | 86 | 1750 | 100 | 375 | 36 | 242 | 100 |
| $[Co(emizco)_2Br_2]{\cdot}H_2O$ | 175 | 13 | 238 | 36 | 1750 | 100 | 375 | 36 | 242 | 100 |
| $Co(NO_3)_2{\cdot}6H_2O$ | 942 | 57 | 602 | 91 | 1750 | 100 | 521 | 50 | 242 | 100 |
| $[Co(emizco)_2(H_2O)_2](NO_3)_2{\cdot}2H_2O$ | 269 | 20 | 311 | 47 | 1750 | 100 | 573 | 55 | 242 | 100 |

*a* = μequiv $e^-$/h x mg Chl

*b* = Activities percentage. Control = 100 %

### Seed germination, respiration and growth

The effects of emizco, its $Co^{2+}$ coordination compounds and the cobalt(II) salts were investigated on seed germination, seed respiration and seedling growth. The following monocotyledonous (*Lolium multiflorum* and *Triticum aestivum)* and dicotyledonous (*Trifolium alexandrinum* and *Physalis ixocarpa*) species were employed.

Emizco inhibited germination and root and shoot growth (Fig. 5). The extent of inhibition increased with concentration up to 200 μM. The pattern of inhibition of germination and shoot development was similar for both monocotyledonous and dicotyledonous species. While *P. ixocarpa* was the most sensitive (Fig. 5) and *Trifolium alexandrinum* was the least (20% germination is still observed at 400 μM). On the other hand, root development for monocotyledonous species was more sensitive to emizco than dicotyledonous plants as shown in Fig. 5. The $I_{50}$ for germination was on the range 27 to 133 μM, and for growth 33 to 102 μM (Table 2). These $I_{50}$ values are in the low range value of many phytochemical compounds tested as reported by Einhellig /27/, therefore emizco is a potent inhibitor for germination and seedling growth. On the activities assayed, emizco showed a similar phytotoxic potency to sorgoleone (10 to 125 μM) /28/.

In order to inhibit seed respiration, higher concentrations of emizco were needed. It was observed that emizco at 400 μM inhibited seed respiration, ranging from 0.0 to 77% (Table 3). The largest effect was observed for *T. aestivum* seeds, where maximum inhibition of the activity was 33% on the first day of imbibitions, and then started to diminish. The other studied species were less affected by emizco (Table 3). Since seed respiration was only partially inhibited, our results suggest that mitochondria might not be a major target of these compounds.

**Emizco**

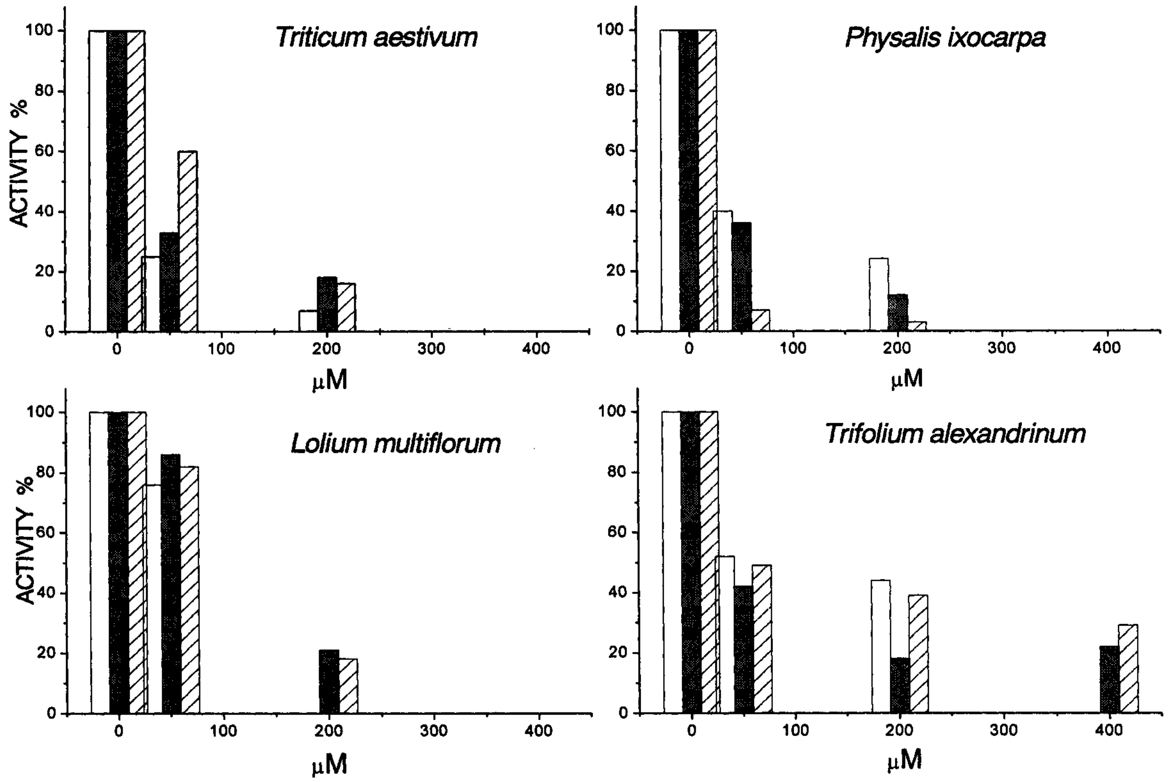

**Fig. 5:** Root length ▭, shoot elongation ■, and seed germination ▨, under emizco effect at 50 μM, 200 μM and 400 μM. Control without compound = 100%.

**Table 2**

$I_{50}$ values in μM for the coordination compounds, emizco and cobalt salts on different seeds. Root growth (*r*), shoot elongation (*s*) and seed germination (*g*). ***i*** means increased growth, elongation, or germination. ne means no effect.

| Compounds | *T. vulgare* | | | *L. multiflorum* | | | *P. ixocarpa* | | | *T. alexandrinum* | | |
|---|---|---|---|---|---|---|---|---|---|---|---|---|
| | *r* | *s* | *g* | *r* | *s* | *g* | *r* | *s* | *g* | *r* | *s* | *g* |
| $[Co(emizco)_2Cl_2]$ | 178 | 269 | 200 | 130 | >400 | 394 | 208 | 270 | 287 | 314 | >400 | 384 |
| $[Co(emizco)_2Br_2]\cdot H_2O$ | >400 | ne | ne | 288 | ne | ne | 178 | 334 | 87 | 246 | 37 | 278 |
| $[Co(emizco)_2(H_2O)_2](NO_3)_2\cdot 2H_2O$ | 355 | 315 | >400 | 135 | ne | ne | 68 | 254 | 369 | 232 | 331 | ne |
| emizco | 33 | 36 | 85 | 102 | 122 | 133 | 41 | 41 | 27 | 78 | 55 | 46 |
| $CoCl_2\cdot 6H_2O$ | 339 | 396 | 400 | 465 | ne | ne | >400 | ne | ne | >400 | >400 | ne |
| $CoBr_2$ | 432 | >400 | >400 | >400 | ***i*** | ***i*** | 322 | 384 | >400 | >400 | ***i*** | >400 |
| $Co(NO_3)_2\cdot 6H_2O$ | 517 | 593 | ne | ne | ***i*** | ne | 400 | ne | ne | 355 | 295 | >400 |

**Table 3**

Effect of metal salts, coordination compounds and emizco at 400μM on seed respiration at 1, 3 and 5 days. Range of rate respiration. Control values in nanoatom•$h^{-1}$•$seed^{-1}$ are 1000 to 1600.

| Compound | *T. vulgare* | | | *L. multiflorum* | | | *P. ixocarpa* | | | *T. alexandrinum* | | |
|---|---|---|---|---|---|---|---|---|---|---|---|---|
| | 1 | 3 | 5* | 1 | 3 | 5* | 1 | 3 | 5* | 1 | 3 | 5* |
| | *Percentage of Control* | | | | | | | | | | | |
| Control | 100 | 100 | 100 | 100 | 100 | 100 | 100 | 100 | 100 | 100 | 100 | 100 |
| $CoCl_2 \cdot 6H_2O$ | 70 | 65 | 39 | 100 | 100 | 30 | 17 | 10 | 25 | 150 | 83 | 75 |
| $[Co(emizco)_2Cl_2]$ | 64 | 75 | 50 | 100 | 100 | 100 | 107 | 50 | 64 | 80 | 100 | 50 |
| $CoBr_2$ | 80 | 81 | 126 | 120 | 100 | 30 | 50 | 30 | 13 | 100 | 100 | 42 |
| $[Co(emizco)_2Br_2] \cdot H_2O$ | 80 | 90 | 55 | 75 | 140 | 133 | 100 | 100 | 55 | 80 | 100 | 58 |
| $Co(NO_3)_2 \cdot 6H_2O$ | 80 | 80 | 75 | 200 | 75 | 20 | 33 | 20 | 25 | 80 | 100 | 50 |
| $[Co(emizco)_2(H_2O)_2](NO_3)_2 \cdot 2H_2O$ | 79 | 75 | 41 | 88 | 80 | 117 | 89 | 79 | 75 | 80 | 80 | 83 |
| emizco | 33 | 40 | 80 | 100 | 100 | 60 | 83 | 54 | 88 | 89 | 40 | 54 |

*1,3,5 days

Phytotoxicity of the coordination compounds: $[Co(emizco)_2Cl_2]$, $[Co(emizco)_2Br_2] \cdot H_2O$ and $[Co(emizco)_2(H_2O)_2](NO_3)_2 \cdot 2H_2O$, was studied. Seed germination, growth (root growth and shoot development) (Table 2) and respiration (Table 3) were either slightly inhibited or non-affected by these compounds, even at high concentrations (400 μM). As an example, Fig. 6 shows the effect of $[Co(emizco)_2Cl_2]$ on seed germination and seedling growth. The latter was less potent than emizco (Fig. 5). The results for the remaining coordination compounds, at 400 μM, are listed in Table 2. $[Co(emizco)_2Cl_2]$ had the highest phytotoxic activity on germination. However, coordination compounds were less potent than the ligand (Table 2). Interestingly, the presence of the metal ion diminished the inhibitory potency of emizco (compare Fig. 5, 6 and Table 3). Therefore, its coordination to $Co^{2+}$ may have diminished emizco phytotoxicity.

Phytotoxicity was concentration-dependent for the coordination compounds, the higher effects occurred at 400 μM (Table 2). Of the evaluated processes, respiration was the least affected by the coordination compounds (Table 3).

Root and shoot development, and seed germination were only inhibited by the coordination compounds at high concentration. Fig. 7 shows that emizco prevents germination and seedling growth, contrary to the effect observed with $[Co(emizco)_2Cl_2]$ that allows germination even at 400 μM, Fig. 8.

Phytotoxicity of the cobalt(II) salts was tested as control in parallel experiments. In general, none of the salts had any effect on germination or seedling growth (Table 2); in some cases it slightly inhibited or stimulated these processes. In Table 3, it can be appreciated that respiration is inhibited by the salts only after five days of treatment; the effect being larger in ***Lolium multiflorum*** and ***Physalis ixocarpa***. This may be due to the presence of the anions.

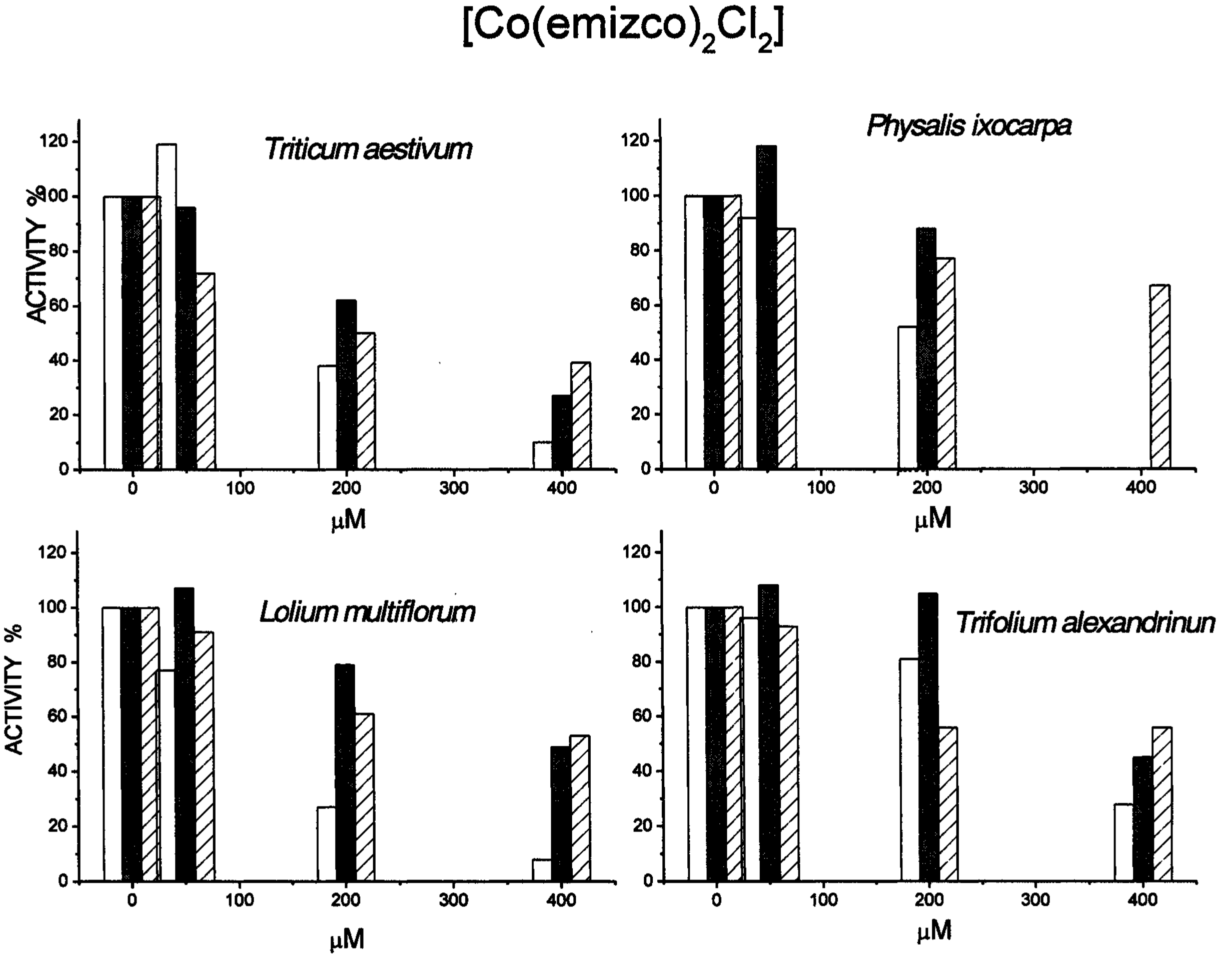

**Fig. 6:** Root length ☐, shoot elongation ■ , and seed germination▨, under $[Co(emizco)_2Cl_2]$ effect at 50 μM, 200 μM and 400 μM. Control without compound = 100%.

Our results show that emizco is a potent phytotoxic compound that inhibits germination, and root and shoot development on monocotyledonous and dicotyledonous species. According to its $I_{50}$ value, emizco is approximately two to three times less active than a standard commercial herbicide, such as methazole (2-(3,4-dichlorophenyl)-4-methyl-1,2,4-oxadiazolidine-3,5-dione) and butaminfos (*o*-ethyl-*o*-(6-nitro-*m*-tolyl)-*sec*-butyl phosphoramidothioate).

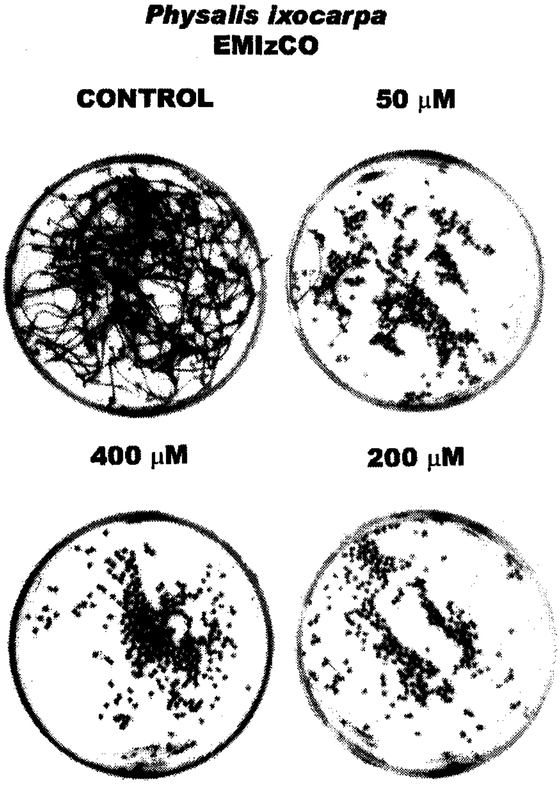

Fig. 7: *Physalis ixocarpa* seeds in Petri dishes in the presence of emizco at 50, 200 and 400 μM.

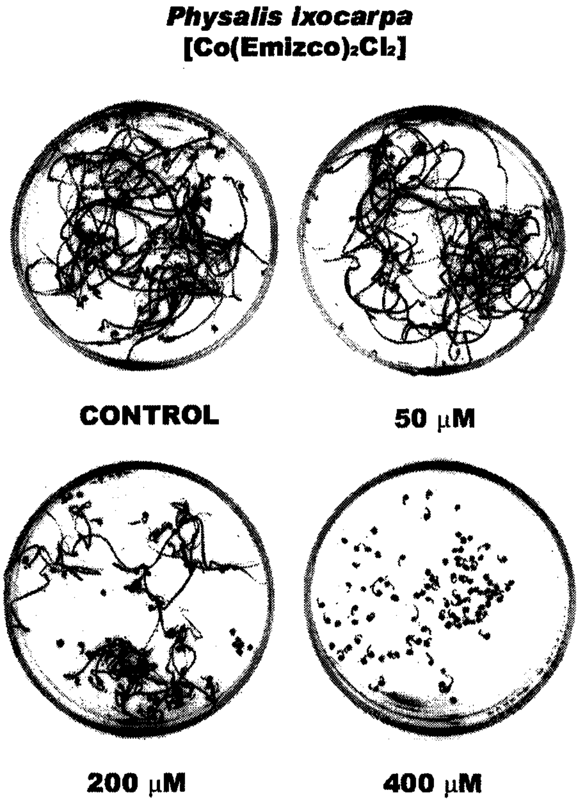

Fig. 8: *Physalis ixocarpa* seeds in Petri dishes in the presence of $[Co(emizco)_2Cl_2]$ at 50, 200 and 400 μM.

### CONCLUSIONS

As previously observed /8/ neither the anions ($Cl^-$, $Br^-$ and $NO_3^-$) nor emizco by themselves contribute to the inhibition of electron transport (Table 1). However, when emizco is bound to cobalt(II), the coordination compounds inhibit electron transport, behaving as more potent Hill reaction inhibitors than the salts, and reducing emizco toxicity. For the coordination compounds there are two inhibition sites on PSII; one of them is also a target for the metal salts. On the other hand, emizco is a potent germination and seedling growth inhibitor, while the metal salts and coordination compounds have no effect.

### ACKNOWLEDGEMENTS

This work was supported by grants DGAPA IN213800 and IN216698. B. King-Díaz acknowledges the fellowship awarded by CONACyT, México. We acknowledge Patricia Fierro for technical support.

### REFERENCES

1. J.E. Bennett, Antimicrobial agents. Antifungal agents. In: *The Pharmacological Basis of Therapeutics.* Goodman and Gilman (Eds.), Pergamon Press Inc., New York. 1990; pp.1169-1181.
2. H. Garaboyes, T.J. Kasper and P. Vaidya, *5-Methyl-4-imidazolecarboxylic acid esters intermediate to cimetidine.* Eur. Pat. Appl. EP 49, 630. US, 1982.
3. J.H. Parsons, D.J. Simpson, P.J. Dudfield and R.G. Hunt, *5-Methyl-4-imidazolecarboxylic acid esters intermediate to acylcyanoazoles as agrochemical fungicides.* Brit. UK Pat. Appl. GB 21, 245, 565. pp. 27, 1990.
4. R. Alonso, J.I. Andrés, M.T. García-López, F.G. De las Heras, R. Herranz, B. Alarcón and L. Carrasco, *J. Med. Chem.* **28**, 834-838 (1985).
5. G. Beck, H. Heitzer, L. Eue and R.R. Schmidt, *Halogenated derivatives of imidazolecarboxylic acids and their use as herbicides.* Bayer A.-G. Eur. Pat. 1979, Appl. 31,086, 1993.
6. F.D. Hess and R.H. Falk, *Weed Sci.*, **38**: 280-288 (1990).
7. A.E. Ceniceros-Gómez, B. King-Díaz, N. Barba-Behrens, B. Lotina-Hennsen and S.E. Castillo-Blum, *J. Agric. Food Chem.*, **47**: 3075-3080 (1999).
8. B. King-Díaz, N. Barba-Behrens, J. Montes-Ayala, S.E. Castillo-Blum, C. Escartín-Guzmán, R. Iglesias-Prieto and B. Lotina-Hennsen, *Z. Naturforsch.*, **53c**: 987-994 (1998).
9. H. Marschner, Function of mineral nutrients in micronutrients. In: *Mineral Nutrition of Higher Plant,* Academic Press Inc. University Printing House, Cambridge, Great Britain, 1988; pp. 313-404.
10. S. Palit, S. Archana and G. Talakder, The effect of cobalt on plants. In: *The Botanical Review,* D.W.M. Stevenson (Ed.), Allen Press Inc. Kansas **60**: 149-181 (1994).
11. V.I. Volkorezov, *Uch. Zap. Gor'k Univ.*, **90**: 114-117 (1968).
12. S.M. Siegel, *Water Air Soil Pollut.*, **8**: 293-304 (1977).

13. H. Tuppy and W. Sieghart, *Monatsh. Chem.*, **104**: 1433-1443 (1973).
14. B.C. Tripathy, B. Bathia and P. Mohanty, *Biochim. Biophys. Acta.* **722**: 88-93 (1983).
15. N. Mohanty, Y. Vass and S. Demeter, *Physiol. Pl.*, **76**: 386-390 (1989).
16. S. Saha, R. Ouuitrakul, S. Izawa and N. Good, *J. Biol. Chem.* **246**, 3204-3209 (1971).
17. B. Lotina-Hennsen, J. Roque-Resendiz M. Jiménez and M. Aguilar, *Z. Naturforsch.* 46c, 1772-1780 (1991).
18. H.H. Strain, B.T. Coppe and M. Sve, *Methods. Enzymol.* **23**, 452-466 (1971).
19. R. Dilley, *Methods Enzymol.* 24, 68-74 (1972).
20. M.R. Calera, F. Soto, P. Sánchez, R. Bye, B. Hernández, A.L. Anaya, B. Lotina-Hennsen and R. Mata, *Phytochemistry.* **40**, 419-425 (1995).
21. J.F. Allen and N.G. Holmes, Electron transports partial reactions. In: *Photosynthesis, Energy Transduction. A Practical Approach* (M.F. Hipkins and N.R. Baker, Eds.). IRL Press, Oxford, U.K., 1986; pp 119-128.
22. R.J. Strasser, A. Srivastava and Govindjee, *Photochem. Photobiol.* **61**, 32-46 (1995).
23. F.A. Macias, Allelophathy in the search for natural herbicide models. In: *Allelopathy: Organisms, Processes and Applications*, K.M. Inderjit, M. Dakshini and F.A. Einhellig (Eds.), American Chemical Society: Washington, DC, USA, 1995; pp. 311-329..
24. M.P. Fialon, E. García-Baéz, N. Andrade-López, G. Osorio-Monreal, G. Canseco Melchor, I. Vásquez-Montes, N. Barba-Behrens and R. Contreras, *Heteroatom Chem.*, **10**, 577-584 (1999).
25. K. Kurdziel, T. Glowiat and J. Jezierska, *Polyhedron*, **20**, 3307-3313 (2001).
26. A.V.P. Lever, *Inorganic Electronic Spectroscopy*, 2nd ed., Amsterdam: Elsevier, 1984; pp. 479-520.
27. F.A. Einhellig, Mechanism of action of allelochemicals in allelopathy. In: *Allelopathy: Organisms, Processes and Applications*; K.M. Inderjit, M. Dakshini and F.A. Einhellig (Eds.), American Chemical Society: Washington, DC, USA, 1995; pp. 97-115.
28. F.A. Einhellig and Y.F. Souza, *J. Chem. Ecol.*, **18**: 1-11 (1992).

